# Relationship between shift work, night work, and subsequent dementia: A systematic evaluation and meta-analysis

**DOI:** 10.3389/fneur.2022.997181

**Published:** 2022-11-07

**Authors:** Zhen-Zhi Wang, Zhen Sun, Mei-Ling Zhang, Kang Xiong, Feng Zhou

**Affiliations:** ^1^The First Clinical Medical College of Shaanxi University of Traditional Chinese Medicine, Xianyang, China; ^2^Hengyang Medical School, University of South China, Hengyang, China; ^3^The Affiliated Hospital of Shaanxi University of Traditional Chinese Medicine, Xianyang, China

**Keywords:** shift work, night work, dementia, risk factor, systematic evaluation, meta-analysis

## Abstract

**Background:**

The association between shift work, night work, and the risk of dementia are conflicting in the literature.

**Objectives:**

This study was performed to obtain evidence on the potential relationship between shift work, night work, and dementia.

**Methods:**

To investigate the link between shift work, night work, and dementia, we systematically searched PubMed, Embase, and Web of Science from inception to January 1, 2022. The eligibility of the retrieved records and extracted data were independently reviewed by two researchers. The Preferred Reporting Items for Systematic Evaluations and Meta-Analyses (PRISMA) statement was followed. STATA 16.0 was used to conduct the meta-analysis.

**Results:**

A total of four studies involving 103,104 participants were included in this meta-analysis. Pooled results indicated that night shift workers had a 12% increased risk of dementia compared to subjects without night work (HR = 1.12, 95% CI 1.03–1.23, *P* = 0.094). Shift work was not significantly associated with dementia risk (HR: 1.09, 95% CI: 0.83–1.43, *P* = 0.546), but the effect of shift work on dementia risk appeared to increase with age, with a correlation observed among workers older than 50 years (HR = 1.31; 95% CI: 1.03–1.68, *P* = 0.030).

**Conclusion:**

The data presented in our study suggest that night work may be a risk factor for dementia. More prospective studies with objective exposure measurements are required to further confirm this result.

**Systematic review registration:**

https://doi.org/10.37766/inplasy2022.6.0079, identifier: INPLASY202260079.

## Background

Shift work includes work schedules that go beyond the typical “9 to 5” working day and often include early starts, compressed work weeks with 12-h shifts, and night work. With the rapid economic growth of modern society, shift and night work is becoming increasingly widespread in various industries such as food production, entertainment, aviation, healthcare, and transportation (1–3). However, the disruption of circadian rhythms caused by shift and night work poses a significant challenge to public health, increasing the risk of cardiovascular disease, cancer, diabetes, and neuropsychiatric disorders (4–7), even causing specific mortality (8). Recent studies have shown that encountering conditions such as night shift work schedules can lead to lack of sleep, poor sleep quality, reduced recovery, and increased physical stress (9, 10).

Since the beginning of the 21st century, population aging has become a common problem in countries around the world (11). With the increase in human life expectancy and the intensification of population aging, neurodegenerative diseases such as Alzheimer's disease, PD, Huntington's disease, and frontotemporal dementia are on the rise every year, posing a serious threat to human health (12). Not only do they cause severe suffering to patients and their families, but they also pose a heavy burden on healthcare services and society as a whole. For example, the annual cost of medical and long-term care for people with Alzheimer's disease in the United States is estimated at USD 259 billion (13). Therefore, it is particularly necessary and urgent to achieve antecedent research and exploration of neurodegenerative diseases, focusing on the earlier stages of cognitive decline. The characteristic early components of cognitive decline have been unclear so far (14); however, recent studies have revealed that the circadian system regulates many physiological processes in the body, such as sleep-wake cycles, metabolism, inflammatory responses, and gene transcription for oxidative stress (15). Animal studies have shown that in the central nervous system, circadian rhythm disturbances can lead to neuroinflammatory responses, oxidative stress, and neuronal death (16), suggesting a strong link between circadian rhythms and central nervous system-related diseases. Some longitudinal studies with long follow-up periods (5–41 years) have similarly reported greater cognitive decline and increased risk of dementia in people with circadian rhythm disorders (CRDs) (including shift work) compared to those without CRDs (17–19). Therefore, we aim at conducting a meta-analysis by summarizing the existing evidence to systematically evaluate the association between shift/night work and risk of dementia.

## Methods

### Search strategy

The Preferred Reporting Items for Systematic Evaluations and Meta-Analyses (PRISMA) statement was followed when performing the systematic evaluation and meta-analysis (20). We comprehensively searched PubMed, Embase, and Web of Science for studies from the build date of each database to January 1, 2022; our search strategy was not restricted by language or year of publication. The following terms were used to construct the search strategy: “night shift work,” “night work,” “shift work,” “shiftwork,” “dementia,” and “Dementias.” The search method was not restricted by publication type. The search strategies are described in Supplementary material. All authors then manually searched references for inclusion in the articles to add further relevant studies.

### Study selection

Title and abstract screening were carried out by two independent authors' reviews, followed by full-text screening. The studies included in the meta-analysis met all of the following criteria: (a) inclusion in observational case-control studies or cohort studies; (b) the main exposure of study was night/shift work, and the outcome was dementia risk; (c) studies reporting effect estimates with corresponding 95% confidence intervals (CIs) or providing relevant data to calculate crude hazard ratios (HRs) for the association between shift work and dementia. The excluded studies met one of the following criteria:(a) conference abstracts, reviews, or letters; (b) pilot studies; (c) case report, systematic reviews and meta-analyses; (d) duplicate publications; and (e) literature for which full text was not available.

### Data extraction

Two authors independently extracted the relevant data from the final included studies, and when differences of opinion arose, the consensus was reached through discussion. If a consensus could not be reached, the disagreement was resolved by consultation with relevant experts. we used pre-designed Microsoft Excel data tables to extract basic information about the selected studies after they had been selected, including first author details such as occupation, country, and age; study year; definition of exposure; and study duration. We also extracted variables impacting the association between night shift work and dementia.

### Quality assessment

The literature was evaluated by two authors using the New-castle-Ottawa Scale (NOS), a standard for evaluating the quality of literature. The cohort studies consisted of eight items in three areas of study population selection, comparability between groups, and measurement of outcome. The case-control studies included eight components in three areas: research population selection, group comparability, and exposure factor assessment. If the prerequisites were satisfied, a score of one out of nine was given, with a score of seven being deemed high-quality literature (21).

### Statistical methods

Meta-analysis of effect models was performed using STATA 16.0 software. The hazard ratios (HRs) were pooled to examine the relationship between night/shift work and dementia risk. I^2^ and Q tests were used to assess heterogeneity. If I^2^ was ≤50% and *P* ≥ 0.05 the heterogeneity across the included literature was low, and a fixed-effects model was selected for analysis. If I^2^ was >50% and *P* < 0.05, the heterogeneity across the included literature was high, and a random-effects model was used. Sensitivity analyses were conducted using a literature-by-literature exclusion method, and consistent conclusions after literature-by-literature exclusion suggested that the results were robust. Bias was evaluated using the Begg's and Egger's tests, and a P >0.05 indicated the absence of publication bias.

## Results

### Literature search

**Figure 1:**
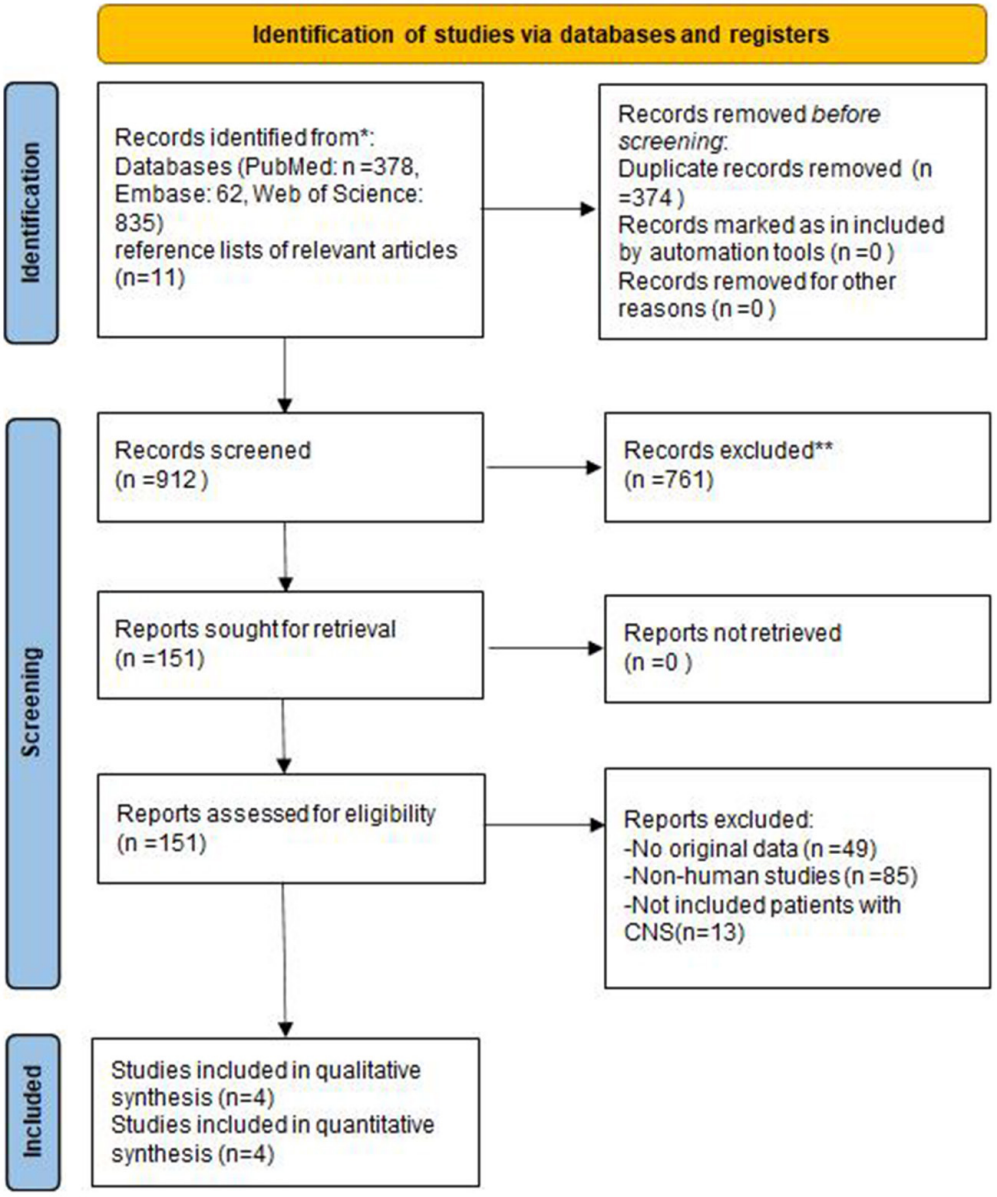
Flow diagram of the study selection process.

A flow chart of the systematic search and inclusion of studies in this meta-analysis were shown in Figure 1. Initially, 1,286 publications were identified using a complete systematic search of PubMed, Web of Science, and Embase, of which 374 were found to be duplicates. 761 publications were eliminated from the remaining investigations because they were either reviews, systematic reviews, animal trials, or meeting abstracts and did not fit the inclusion requirements. Following a full-text review, 147 items were determined to be unsuitable for recording. Eventually, a total of four articles were included in this meta-analysis (7, 19, 22, 23).

### Study characteristics

The period of publication of the selected articles was from 2017 to 2020. Of the four included studies, all were prospective cohort studies (19, 22, 23). Three studies were conducted in Denmark (7, 22, 23), and one in Sweden (19). One study included only female nurses (7), and the remaining few included multiple occupations. The included studies used the world health organization's international classification of diseases (ICD) criteria (7, 19, 22, 23). The following ICD codes were used to identify dementia diagnoses collected from National Health Registers using Personal Identification Numbers (PIDs) (7), the Danish psychiatric registry, and the Psychiatric Register (23). The definition of shift and night work varied between studies, with the definition of exposure mainly including start and end times. One study defined night work as work between 23:00–7:00 (7), whereas two did not mention specific times (19, 23), with varying definitions of shifts. None of the cohort studies included participants with a diagnosis of dementia at baseline. Table 1 describes the main characteristics of the included studies.

**Table 1:** Main characteristics of the included studies.

**Study, year**	**Design**	**Country**	**Sources of participants**	**Number of participants (analyzed)**	**Occupation**	**Mean age years**	**Shift/night work description**	**Exposure definition**	**Definition of dementia**	**Unexposed definition**	**Exposure intensity (mean shift/night work per..)**	**Follow-up (years)**	**Adjustment variables**	**Subgroup analysis**
Nabe-Nielsen et al. (23)	Cohort, prospective	Denmark	Copenhagen Male Study	Total:4,731 Case: 1,011 control:3720	Various	40–59	Working outside normal daytime hours, nightshift work	Fixed evening and night work, roster work, and ordinary 3-shift work	Dementia:: ICD-8 and ICD-10	No shift work at any time point	Not reported	Mean:17.8	Psychological stress, hours of sleep per night, cardiovascular disease, smoking, exercise, alcohol drinking, body mass index, blood pressure	Country, Occupation
Jørgensen et al. (7)	Cohort, prospective	Denmark	Danish Nurse Cohort	Night:total: 12,870; case: 1,048; control:11,822 shift: total: 15,921; case: 4,099; control:11,822	Nurses	≥44	Rotating shift work refers to a work schedule that rotates between at least two of the following: day, evening, and night work.	Night shift: from 23:00 to 07:00. shift work: rotating between at least two of the following shifts: day, evening, and night work	Dementia: ICD-8:290.09–290.19; 293.0919; ICD-10:F00-F03; G30; G31.8-9; ATC code: N06D	persistent day workers	have worked the same shift type for at least 6 years	Mean: 12.5	Work hours, BMI, Smoking, use of hormone replacement therapy, coffee	Country, Occupation, Design, Age at baseline, Preexisting cardio-metabolic disease, Work hours, Used sleep medication
Bokenber ger et al. (19)	Cohort, prospe ctive	Sweden	Swedish Twin Registry	STR-1973:13283 SALT:41199	Various	STR-1973:37.8 SALT:58.5	Shift work and night work	Not reported	Dementia:ICD-8:290, 293.0, 293.1; ICD-9:290.0, 290.1, 331.0, 290.4, 290.8, 290.9, 294.1, 331.1, 331.2, 331.9; ICD-10:G30, F01, F02, F03, F05.1, G31.1, G31.8A; ATC codes:N06D	Dayworkers	Not reported	Median: 41.2	Age, sex, education, diabetes, CVD, and stroke	Occupation, Exposure duration, STR-1973:shift work, shift work duration SALT: shift work, shift work duration
Nabe-Nielsen et al. (22)	Cohort, prospe ctive	Denmark	Central Population Register	Night: total:3,224 case:396 control:2,828 shift: total:2,943 case:115 control:2,828	Various	18–59	Shift work: variable was categorized into ‘day work' shift work without/with unknown exposure tonight work and night shift work, night shift work: three-shift workers and permanent night workers.	Shift work: three-shift work with rotating shifts, three-shift work with variable shifts, permanent night work night shift :three-shift workers and permanent night	Dementia:ICD-8: 290.09–19,290.11,293.09–19; ICD-10: F00.0–9, G30.0–9, F01.0–9, F02.0, G31.8, G31.9	Day work	Not reported	Mean: 9.8	Sex, age, vocational education, Smoking, hypertension, BMI, alcohol, education, psychosocial work factors, lifestyle-related cardiovascular risk factors	Country, Occupation

The four included studies were all considered to be of high quality, with scores ranging from 7 to 9 (Table 2).

**Table 2:** Quality assessment of included studies.

**Study (cohort)**	**Representative of the exposed cohort**	**Selection of non-exposed cohort**	**Ascertainment of exposure**	**Outcome not present before study**	**Comparability**	**Assessment of outcome**	**Follow-up long enough**	**Adequacy of follow up**	**Quality score**
Nabe-Nielsen et al. (23)	*	*	*	*	**	*	*	*	9
Jørgensen et al. (7)		*	*	*	**		*	*	7
Bokenberger et al. (19)	*	*	*	*	**	*	*	*	9
Nabe-Nielsen et al. (22)	*	*	*	*	**	*	*	*	9

### Association between shift work and risk of dementia

**Figure 2:**
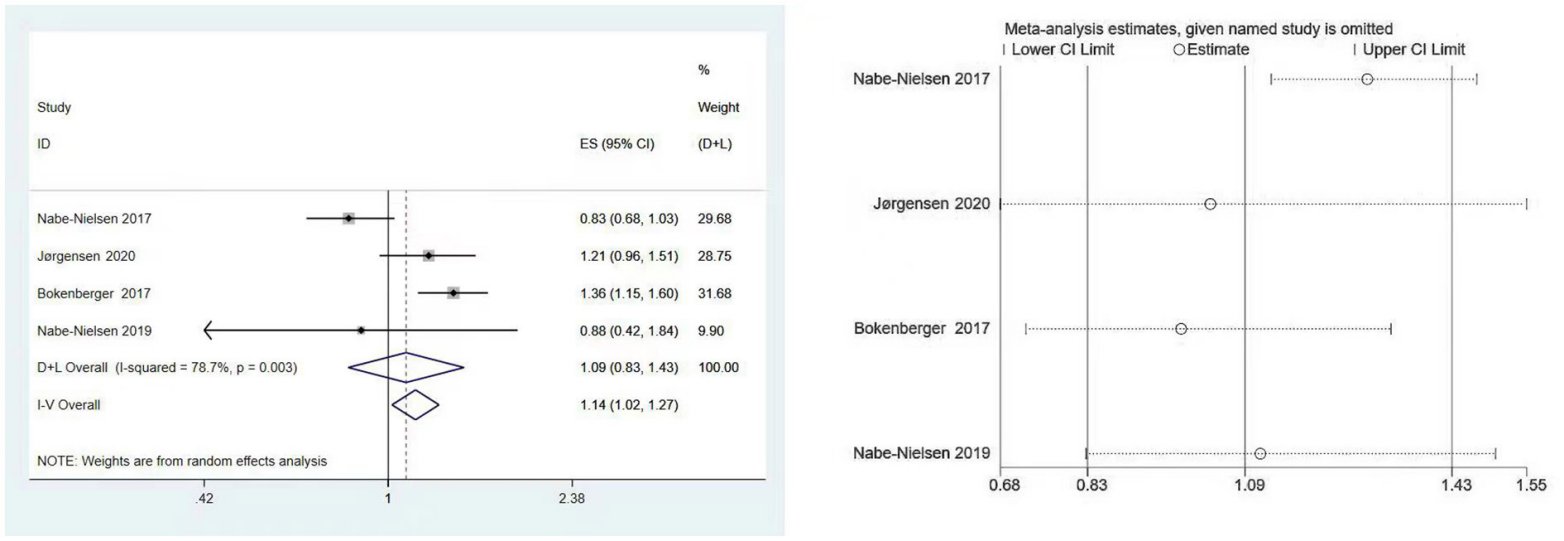
Forest plot and sensitivity analysis of the relationship between shift work and dementia risk.

Four studies involving 40,130 subjects (7, 19, 22, 23) assessed the association between shift work and the subsequent onset of dementia. Significant heterogeneity was observed (I^2^ = 78.7%, P [heterogeneity] = 0.003), as the random-effects model was used. There was no statistically significant link between shift work and dementia, according to the pooled result (HR: 1.09, 95% CI: 0.83–1.43, *P* = 0.546) (Figure 2).

To see if specific study characteristics influenced results, we performed subgroup analyses by country (Denmark, Sweden), occupation (nurses or mixed), years of shift (<10 vs. >10), age at baseline (<50 vs. >50 years), whether studies were adjusted for pre-existing cardiometabolic disease (yes or no) (Table 3).

**Table 3:** Subgroup analysis of the association between shift work and risk of dementia.

**Subgroup**	**N**	**I^2^ (%)**	**P for heterogeneity**	**HR**	**Pooled model**	**Statistically significant *p*-value**
**Country**						
Denmark	2,3595	65.9	0.053	0.98(0.73–1.33)	Random-effects model	0.908
Sweden	13,283	NA	NA	1.36(1.15–1.60)	NA	0.000
**Occupation**						
Nurses	15,921	NA	NA	1.21(0.96–1.51)	NA	0.099
Mixed	20,957	85.4	0.001	1.03(0.68–1.55)	Random-effects model	0.886
**Years of shift**						
<10 y	1,687	NA	NA	1.32(1.09–1.60)	NA	0.005
>10 y	571	NA	NA	1.45(1.11–1.90)	NA	0.007
**Age**						
<50 y	10,947	NA	NA	0.86(0.50–1.58)	NA	0.607
>50 y	7,945	NA	NA	1.31(1.03–1.68)	NA	0.030
**Pre-existing cardio-metabolic disease**						
No	16,712	NA	NA	1.21(0.95–1.54)	NA	0.122
Yes	1,892	NA	NA	1.34(0.71–2.51)	NA	0.364

When country subgroups were considered, pooled results from Denmark showed that the shift work had no effect on risk of dementia (HR = 0.98; 95% CI: 0.73–1.33, *P* = 0.908) (7, 22, 23), while the study from Swedish showed a statistically significant association between shift work and dementia (HR = 1.36; 95% CI: 1.15–1.60, *P* < 0.001) (19).

In terms of the effect of age, Jørgensen et al. (7) found that older (>50 years) shift workers had a dementia risk estimate of 1.31 (HR = 1.31; 95% CI: 1.03–1.68, *P* = 0.030), while people younger than 50 years was not significant (HR = 0.86; 95% CI: 0.50–1.58, *P* = 0.607). Nabe-Nielsen et al. (23) found that the incidence of dementia increased by 63% every 5 years with increasing age (HR = 1.63; 95% CI: 1.50–1.78, *P* < 0.001). Bokenberger et al. and Nabe-Nielsen et al. did not assess the effect of age on the incidence of dementia (19, 22).

In studies that ascertained exposure to occupation, length of sleep, and pre-existing cardio-metabolic disease, no obvious effect on the association between shift work and dementia was observed (7, 19, 22, 23) (Table 3).

### Evaluation for publication bias and sensitivity analysis

The results of Begg's and Egger's test indicated that there was no significant risk of publication bias for the included studies (*P* = 0.734 and 0.656, respectively). Furthermore, according to sensitivity analysis, the association between shift work and dementia risk was relatively stable (Figure 2).

### Association between night shift work and risk of dementia

**Figure 3:**
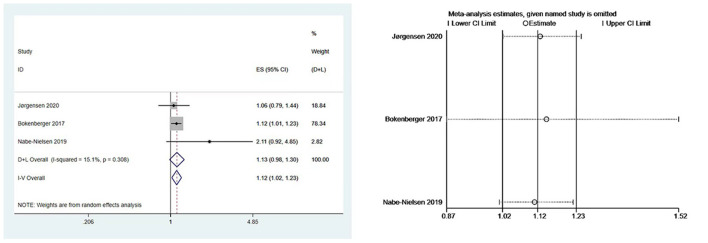
Forest plot and sensitivity analysis of the relationship between night work and dementia risk.

We performed meta-analyses and calculated pooled effect estimates for three studies (7, 19, 23) that included 63,034 subjects and detected no substantial heterogeneity (I^2^ = 15.1%, *P* [Heterogeneity] = 0.308) in the included studies. The fixed-effects models were used to pool multivariate-adjusted HRs across studies to determine the association between night shift work and the risk of dementia (Figure 3). The overall results showed that night shift work may be associated with an increased risk of dementia (HR = 1.12, 95% CI = 1.02–1.23, *P* = 0.094).

Our subgroup analysis was conducted by country (Denmark and Sweden), occupation type (nurses vs. mixed), and years of night shift (<10 and >10 years). The results are listed in Table 4.

**Table 4:** Subgroup analysis of the association between night shift and risk of dementia.

**Subgroup**	**N**	**I^2^**	**P for heterogeneity**	**HR**	**Pooled model**	**Statistically significant *p*-value**
**Country**						
Denmark	15,813	57.1	0.127	1.33(0.71–2.52)	Random-effects model	0.374
Sweden	41,199	NA	NA	1.12(1.01–1.23)	NA	0.024
**Occupation**						
Nurses	12,870	NA	NA	1.06(0.79–1.44)	NA	0,704
Mixed	44142	54.5	0.138	1.34(0.77–2.33)	Random-effects model	0.307
**Years of night shift**						
<10 y	6,513	NA	NA	1.13(0.99–1.28)	NA	0.062
>10 y	5,886	NA	NA	1.10(0.97–1.25)	NA	0.120

N, number; HR, hazard ratio.

Our subgroup analysis showed that, when grouped by country, the HR of night shift work in Denmark and Sweden was 1.33 (95% CI: 0.71–2.52, *P* = 0.374) (7, 22, 23) and 1.12 (95% CI: 1.01–1.23; *P* = 0.024) (19), respectively. When grouped by occupation (7, 19, 22, 23), the pooled HR was 1.06 (95% CI: 0.79–1.14; *P* = 0.704) for the nurse group and 1.34 (95%CI: 0.77–2.33; *P* = 0.307) for the mixed occupation group.

### Evaluation for publication bias and sensitivity analysis

Begg's and Egger's tests yielded P values of 1.000 and 0.552, respectively, showing that there was no publication bias. Due to the minimal number of papers covered, funnel plots were not created. Moreover, a sensitivity analysis was carried out by removing one study at a time (Figure 3), the results remained stable.

## Discussion

### Principal findings

According to the primary pooled results, we found that there was no statistically significant association between shift work and the risk of dementia (HR = 1.09, 95% CI: 0.83–1.43, *P* = 0.546), but night shift workers have a 12% increased risk of developing dementia (HR = 1.12, 95% CI: 1.02–1.23, *P* = 0.094). We conducted a subgroup analysis to assess whether specific study characteristics had an impact on the link between shift/night work and dementia. Despite the small number of studies included, the findings of this analysis may be regarded as a reliable estimate based on high-quality research with over 900,000 participants. These findings add to prior evidence of a potentially detrimental effect of night work on health and longevity.

### Comparison with other reviews

A systematic review published in 2021 due to the limited number of studies included showed no conclusions can be drawn about the relationship between shift work and dementia (24). Compared to their study, our meta-analysis can not only qualitatively analyze the association between shift work, night shift and dementia, but also quantitatively synthesize the hazard ratio between them, and draw comprehensive conclusions, so as to provide scientific evidence that reduces bias as much as possible and approaches the real risk. The findings in our study regarding overall dementia risk were consistent with theirs, and night shift workers had a 12% increased risk of dementia compared to subjects without night shifts (HR = 1.12, 95% CI 1.02–1.23). Our results do not support that shift work is associated with a risk of dementia. (HR: 1.09, 95% CI: 0.83–1.43). Unfortunately, most studies did not provide the duration and intensity of shift work and night work, so we have insufficient data to conduct a more detailed study. Geographical variation and genetic diversity may play a role in the correlation between shift work, night work and dementia.

### Effect of shift work and night work on dementia

The prevalence of dementia is increasing at an alarming prevalence in the aging population. Alzheimer's disease accounts for 60–80% of all cases of dementia and is the most common subtype of dementia (25). It is estimated that the global prevalence of dementia in people aged 65 and over is as high as 7%, with a higher prevalence (8–10%) in developed countries due to the longer life expectancy of the population. (26). Aging, genetic characteristics, and systemic vascular disease are the main risk factors for the development of dementia (27).

There is an established study that confirms that sundowning in dementia and the sleep/wake cycle in delirium patients are influenced by circadian rhythms. Changes in daily routine during night work and shift work can disrupt the body's normal circadian system and lead to a variety of hormonal and metabolic disruptions that can negatively affect physical and mental health (28). One of the pathogenic mechanisms of dementia is the aggregation and accumulation of β-amyloid (Aβ) and tau proteins. Recent evidence from a study by Musiek et al. suggests that sleep rhythm disturbances appear to exacerbate Aβ pathology in mice, and a similar effect may occur in humans as well (29).

A Swedish study (19) showed that the HR for dementia was associated with night work and shift work, and longer exposure to shift/night work appears to be associated with a higher incidence of dementia. In addition, Bokenberger et al. conducted a partial study on the apolipoprotein E (APOE e4) genotype, illustrating that the increased risk of dementia in individuals exposed to shift work and night work lasts 20 years longer in APOE e4 carriers compared to day workers. The incidence of dementia is influenced by genotype; however, other studies (7, 19, 22) have failed to consider genotype-related effects, which may be a factor contributing to the differences in our pooled results.

Jørgensen et al. found (7) that night and shift work did not affect the incidence of dementia compared with fixed day work when any duration of the shift was not considered. They did, however, find a strong relationship between permanent night shift employment for >6 years and the risk of dementia, suggesting that persistent night shift work may increase dementia risk. However, this estimate is based on a tiny number of occurrences. Nonetheless, the increased risk of dementia observed in long-term night shift workers are consistent with the evidence of putative biochemical pathways between sleep and dementia. The findings of this study, which show that night-shift workers have higher dementia risk, are similar to those of a Danish study by Nabe-Nielsen et al. (23), in which higher risk of dementia were observed in permanent night shift workers in the Danish Work Environment Cohort Study (HR = 3.25; 95% CI: 1.35–7.83), consistent with the estimates of Jørgensen et al. (HR = 2.32; 95% CI: 1.32–4.09). In a research of men in Copenhagen, no evidence of a link between midlife shift employment (defined as shift work or regular night work) and dementia incidence over 40 years was discovered (22). The disparities in results between Jørgensen et al. and the Copenhagen study could be explained by major variances in the study populations, including birth cohort and occupational characteristics.

In our age subgroup analysis, the Swedish study found (23) that age may be an important influencing factor in the association of shift and night work with dementia, with older subjects having a higher incidence of dementia than younger subjects and that the differences in the association between shift work and dementia across regions may also be attributed to older age of the Swedish subgroup of participants in our study. Overall, it is generally viewed that the psychological stress induced by the disruption in the circadian rhythm due to night shift work has been suggested to have an adverse impact on brain structures involved in higher cognitive functions (30–33). Shift work may include some non-night shifts as the exposure (34), which may be one of the explanations for this non-significant association for shift work with risk of dementia in current study. Furthermore, putative explanations might be that, most often, permanent night shift workers keep a desynchronized circadian rhythm, with awakening during normal sleeping periods (35). This desynchronized circadian rhythm might affect sleep quality involving chronical disrupted sleep (36, 37). Indeed, day sleeps have typically been found to be considerably shorter than normal night sleeps, because of multiple reasons including increased environmental noise, light levels, conflicting social, and family demands (38). In a word, permanent night shifts might be the poor schedule for workers in terms of dementia.

However, more comprehensive and detailed studies are required to validate our findings. Notably, our pooled risk estimates were heavily influenced by the Swedish cohort study (23), which did not adjust for alcohol consumption, body mass index, or tobacco use, and all potentially relevant confounders consistently varied within each cohort in exposed and unexposed subjects. Especially, shift and night workers may have different lifestyles and behaviors (smoking and/or obesity) (39, 40), and dementia prevalence may vary depending on region and heredity (41, 42).

### Limitations

Despite the originality of our results, there are some limitations to our study. First, in the case of shift night work, there is insufficient data to conduct a more detailed study if only the present shift work schedule is considered, rather than the regularity/irregularity of the schedule, as well as the duration and intensity. Second, with the exception of Bokenberger et al. who did not give an explicit definition of shift night work, the authors of the other studies defined shift night work differently because of different study populations (e. g., specific groups of workers or general working populations), different methods of exposure measurement (e. g., job exposure matrices or questionnaires) and different definitions of exposure and non-exposure. This shortcoming may give rise to heterogeneity between studies and hinder the identification of reliable risk factors. Thirdly, there may still be confounding factors that cannot be controlled, such as the quality of the time worked or differences in rest periods during shifts. This may have an impact on the results as the number and type of confounding factors considered in each study differ. Finally, the original studies included in this paper are mostly from the Nordic region and are underrepresented. The results obtained in this review must be interpreted with caution due to the lack of studies from other parts of the world.

## Conclusion

In conclusion, the findings of our investigation revealed that night work may be a risk factor for dementia, with no statistically significant association between shift work and the risk of dementia. Due to the absence of detailed data on duration and intensity of shift and night work included in this meta-analysis, these findings should be regarded with care. More high-quality prospective cohort studies are needed to obtain valid and reliable data. Furthermore, detailed assessments of lifetime work schedules are warranted.

## Data availability statement

The original contributions presented in the study are included in the article/Supplementary material, further inquiries can be directed to the corresponding author.

## Author contributions

Z-ZW and ZS developed the protocol, participated in the literature search, extracted data, and drafted the manuscript. M-LZ was responsible for the analysis and interpretation of the data. KX contributed to statistical expertise. FZ supervised. All authors contributed to the article and approved the submitted version.

## Funding

This study was supported by the National Natural Science Foundation of China (81873387) and the National Key Talents Project of Traditional Chinese Medicine (2019). This study was also supported by the foundation of the training Project for Young and Middle-aged Science and Technology leaders in Xianyang (Major Scientific and Technological Innovation Project) (2019k01-52) and the training Program of Shaanxi University of Traditional Chinese Medicine (2017SZKY-018). The funding agents play no role in study design, data collection, and data analyses.

## Conflict of interest

The authors declare that the research was conducted in the absence of any commercial or financial relationships that could be construed as a potential conflict of interest.

## Publisher's note

All claims expressed in this article are solely those of the authors and do not necessarily represent those of their affiliated organizations, or those of the publisher, the editors and the reviewers. Any product that may be evaluated in this article, or claim that may be made by its manufacturer, is not guaranteed or endorsed by the publisher.

## Abbreviations

PRISMA: Preferred Reporting Items for Systematic Evaluations and Meta-Analyses
CI: confidence interval
NOS: Newcastle-Ottawa scale
DA: dopamine
HR: hazard ratio
ICD: International Classification of Diseases
ATC: Anatomical Therapeutic Chemical Classification System
NPR: National Patient Register.
