# Supplementary material for: Relationship between shift work, night work, and subsequent dementia: A systematic evaluation and meta-analysis
Source: Front Neurol. 2022 Nov 7;13:997181. doi: 10.3389/fneur.2022.997181 (PMC9677942; doi:10.3389/fneur.2022.997181)
Supplement: Supplementary file 1 [file Data_Sheet_1.docx]

**PubMed：378**

(("Dementia"[Mesh]) or Dementias or Amentia or Amentias or Senile Paranoid Dementia or Dementias, Senile Paranoid or Paranoid Dementia, Senile or Paranoid Dementias, Senile or Senile Paranoid Dementias or Familial Dementia or Dementia, Familial or Dementias, Familial or Familial Dementias) or (("Alzheimer Disease"[Mesh]) or Alzheimer Dementia or Alzheimer Dementias or Dementia, Alzheimer or Alzheimer's Disease or Dementia, Senile or Senile Dementia or Dementia, Alzheimer Type or Alzheimer Type Dementia or Alzheimer-Type Dementia (ATD) or Alzheimer Type Dementia (ATD) or Dementia, Alzheimer-Type (ATD) or Alzheimer Type Senile Dementia or Primary Senile Degenerative Dementia or Dementia, Primary Senile Degenerative or Alzheimer Sclerosis or Sclerosis, Alzheimer or Alzheimer Syndrome or Alzheimer's Diseases or Alzheimer Diseases or Alzheimers Diseases or Senile Dementia, Alzheimer Type or Acute Confusional Senile Dementia or Senile Dementia, Acute Confusional or Dementia, Presenile or Presenile Dementia or Alzheimer Disease, Late Onset or Late Onset Alzheimer Disease or Alzheimer's Disease, Focal Onset or Focal Onset Alzheimer's Disease or Familial Alzheimer Disease (FAD) or Alzheimer Disease, Familial (FAD) or Familial Alzheimer Diseases (FAD) or Alzheimer Disease, Early Onset or Early Onset Alzheimer Disease or Presenile Alzheimer Dementia)) AND ((“Shift Work Schedule”[Mesh]) or Schedule, Shift Work or Schedules, Shift Work or Work Schedule, Shift or Night Shift Work or Shift Work, Night or Rotating Shift Work or Shift Work, Rotating or night work or shift work)

**Web of science: 835**

(((dementia or Dementias or Amentia or Amentias or Senile Paranoid Dementia or Dementias, Senile Paranoid or Paranoid Dementia, Senile or Paranoid Dementias, Senile or Senile Paranoid Dementias or Familial Dementia or Dementia, Familial or Dementias, Familial or Familial Dementias) or (Alzheimer or Alzheimer Dementia or Alzheimer Dementias or Dementia, Alzheimer or Alzheimer's Disease or Dementia, Senile or Senile Dementia or Dementia, Alzheimer Type or Alzheimer Type Dementia or Alzheimer-Type Dementia (ATD) or Alzheimer Type Dementia (ATD) or Dementia, Alzheimer-Type (ATD) or Alzheimer Type Senile Dementia or Primary Senile Degenerative Dementia or Dementia, Primary Senile Degenerative or Alzheimer Sclerosis or Sclerosis, Alzheimer or Alzheimer Syndrome or Alzheimer's Diseases or Alzheimer Diseases or Alzheimers Diseases or Senile Dementia, Alzheimer Type or Acute Confusional Senile Dementia or Senile Dementia, Acute Confusional or Dementia, Presenile or Presenile Dementia or Alzheimer Disease, Late Onset or Late Onset Alzheimer Disease or Alzheimer's Disease, Focal Onset or Focal Onset Alzheimer's Disease or Familial Alzheimer Disease (FAD) or Alzheimer Disease, Familial (FAD) or Familial Alzheimer Diseases (FAD) or Alzheimer Disease, Early Onset or Early Onset Alzheimer Disease or Presenile Alzheimer Dementia)) AND ((night shift or night shiftwork or night work or nightshift or nightwork or overnight shift or overnight work) or (Shift work or Shiftwork)))

**Embase: 62**

**#15 #7 AND #14 62**

**#14 #10 OR #13 8,349**

**#13 #11 OR #12 7,302**

**#12 'shiftwork' 2,011**

**#11 'shift work'/exp 5,875**

**#10 #8 OR #9 4,779**

**#9 'night shiftwork' OR 'night work' OR 'nightshift' OR 'nightwork' OR 'overnight shift' OR 'overnight work' 2,503**

**#8 'night shift'/exp 3,402**

**#7 3 OR #6 455,824**

**#6 #4 OR #5 278,885**

**#5 'alzeimer disease' OR 'alzeimer s disease' OR 'alzeimers disease' OR 'alzheimer dementia' OR 'alzheimer fibrillary change' OR 'alzheimer fibrillary lesion' OR 'alzheimer neurofibrillary change' OR 'alzheimer neurofibrillary degeneration' OR 'alzheimer neuron degeneration' OR 'alzheimer perusini disease' OR 'alzheimer sclerosis' OR 'alzheimer syndrome' OR 'alzheimer s disease' OR 'alzheimers disease' OR 'cortical sclerosis, diffuse' OR 'dementia, alzheimer' OR 'diffuse cortical sclerosis' OR 'late onset alzheimer disease' 212,349**

**#4 'alzheimer disease'/exp 231,432**

**#3 #1 OR #2 418,717**

**#2 'amentia' OR 'demention' 107**

**#1 'dementia'/exp 418,657**
